# Addiction psychiatry and COVID-19: impact on patients and service provision

**DOI:** 10.1017/ipm.2020.47

**Published:** 2020-05-21

**Authors:** D. Columb, R. Hussain, C. O’Gara

**Affiliations:** 1 Addiction Department, St. John of God Hospital, Stillorgan, Dublin, Ireland; 2 UCD School of Medicine and Medical Specialties, University College Dublin, Dublin, Ireland

**Keywords:** Addiction, alcohol, coronavirus, COVID-19, Ireland, gambling, service provision

## Abstract

The Coronavirus Disease 2019 (COVID-19) pandemic has undoubtedly had a major impact on the provision of physical healthcare in Ireland and worldwide. The mental health impact of this pandemic cannot be underestimated, particularly relating to patients suffering from addiction. Heightened public stress and anxiety levels, increasing isolation and the physical consequences of addiction play a large role in the proliferation and ongoing relapse of substance misuse and behavioural addiction. Service provision is an ongoing challenge not only due to the increasing need for services given the increased mental health burden of COVID-19 but also the restrictions in place in clinical areas to achieve social distancing. The necessary adaptations to service provision provide opportunities for the analysis of current processes used in our addiction unit and the introduction of new processes to our service. The current crisis tests the sustainability of the service to provide the high standard of care required for these patients.

## Introduction

In the weeks and months following the announcement of the novel Coronavirus Disease 2019 (COVID-19) outbreak (World Health Organization, 2019), healthcare resources have been focused predominately on tackling the physical impact of the virus on patients worldwide. The mental health impact of COVID-19 is significant (Torales *et al.* 2020) and we have seen from previous epidemics of this nature that there can be long-term psychiatric sequelae (Mak *et al.* 2009). This current pandemic has seen an increase in fear and fear-induced behaviours in the general public (Dong & Bouey, 2020) with examples of panic buying in Ireland (Bray, 2020) emphasising the current stress levels of the Irish population. There have been calls for increased mental health care during the COVID-19 pandemic (Xiang *et al.* 2020) and this should include patients attending addiction psychiatry services (Murray, 2020).

Our service provides treatment for patients suffering from alcohol dependence syndrome, behavioural addictions such as gambling disorder and other forms of addiction such as prescription and over-the-counter medications. This editorial seeks to highlight some of the impacts that COVID-19 can have on patients in relation to addiction, as well as the impact of COVID-19 on service provision in our facility.

## Impact of COVID-19 on patients in addiction psychiatry

Increased psychological stress levels have been demonstrated in over one-third of the general population during the COVID-19 pandemic (Qiu *et al.* 2020). Over half the population surveyed in one Chinese study rated the psychological impact of the pandemic from moderate-to-severe (Wang *et al.* 2020). Increased stress levels have been shown to play a key role in drug misuse and relapse in addiction (Sinha, 2001). In addition, ongoing increased acute and chronic stress can lead to the development of alcohol and other substance misuse disorders (Brady & Sonne, 1999). In our service, a comparison of numbers of admissions to our inpatient unit this year showed similar levels of admission compared to the same time period in the previous 5 years, with many patients citing the stress related to COVID-19 as a significant factor in their presentations to our service.

From a physical perspective, the use of alcohol and other illicit substances can be detrimental during the COVID-19 pandemic. There is a direct physical effect that inhaled substances can have on the respiratory system in relation to COVID-19 (Vardavas & Nikitara, 2020). Cocaine use can have a damaging effect on the cardiovascular system, further increasing the risk of mortality associated with COVID-19 infection (Marsden *et al.* 2020). The lack of availability of prescription and illicit opioids during this pandemic may lead to decreased tolerance and subsequent overdose (Wakeman *et al.* 2020). The World Health Organization (WHO) has advised that increased alcohol consumption can make a person more vulnerable to adverse health outcomes associated with COVID-19 (World Health Organization, 2020). This is most likely due to the immune suppression seen with chronic alcohol use, and chronic alcohol use has been shown to increase the severity of other known influenza infections (Meyerholz *et al.* 2008). Combined with the diversion of resources in medicine towards the treatment of COVID-19, this could leave addiction patients more vulnerable to the physical consequences of their addiction.

The measures taken by governments globally and in Ireland to stem the COVID-19 pandemic can take a heavy psychological toll on patients living with addiction. Isolation and lack of distraction created by social distancing guidelines can lead to alcohol misuse, relapse of alcohol dependence and potential development of alcohol use disorder (Clay & Parker, 2020). Isolation and boredom are also significant drivers of problem gambling (Ledgerwood & Petry, 2006). Despite the closure of betting outlets and the cessation of major sporting events worldwide, online gambling has seen a significant rise in popularity during the COVID-19 pandemic (Narayan, 2020). There has been heavy promotion of a plethora of other gambling products including online casino games, online slot machines and virtual sports (Davies, 2020). Reports from problem gamblers attending our service highlight the current difficulties they face trying to avoid the ever-increasing lure of these online forms of gambling that do not involve live sports. An example of such is virtual horseracing where gamblers can bet on the outcome of a race between virtual horses on a computer screen. To the casual, controlled gambler, these forms of gambling are not particularly attractive. To problem gamblers, they present an avenue to continue gambling. Many countries have recognised problem gambling as a significant issue during the COVID-19 pandemic, with Latvia imposing a ban on online gambling (iGamingBusiness, 2020) and Portugal considering a similar ban on online casino gambling (Simmons, 2020). The Irish government should consider adopting a similar approach to online gambling during this pandemic, such as imposing a ban on online gambling or placing a temporary betting cap of £50 per day as proposed by members of parliament in the UK (Davies, 2020).

The combination of the increased anxiety and stress due to COVID-19, the isolation caused by the social distancing measures and high availability of alcohol and gambling during this time will contribute to increased mental health difficulties for addiction patients. This, in turn, will increase the demand on services to provide ongoing care to these patients during the pandemic and test the ability of our current systems to provide a service for these patients.

## Impact of COVID-19 on service provision

The COVID-19 pandemic poses significant challenges to service provision. The social distancing guidelines needed to control the spread of the virus have deemed it necessary to evaluate all the processes we use within our service that were once taken for granted. The overarching theme of the changes has been the reduction of close patient contact with staff and other patients where possible. All areas of psychiatry have had to adapt to this crisis with the field of addiction psychiatry being no different.

A large component of the treatment we advise is the use of group-based therapies and the use of peer support groups. Group-based therapies are effective treatments in different types of substance misuse (Luckmann, 2001; Litt *et al.* 2003) and behavioural addiction (Du *et al.* 2010; Jiménez-Murcia *et al.* 2015). These groups reduce the sense of isolation that patients suffering from addiction can experience, enabling patients to witness recovery in others and an opportunity to have peer interactions leading to improved insight into their own condition (Center for Substance Abuse Treatment, 2005). Due to COVID-19 restrictions, we have had to reduce the number of participants within each group and run additional group-based therapies to provide the same level of care to our patients. There is an argument to be made for the use of an internet-based group platform to allow all patients to remain in the group and negate the risk associated with COVID-19 in large group settings. While group-based telepsychiatry can have similar clinical efficacy to traditional group settings (Chakrabarti, 2015), providing the necessary equipment and secure software to implement this intervention in a short period of time is currently not possible in our service. Aside from the increased demands these modifications place on our addiction counsellors to deliver effective treatment, separating groups can disrupt the pre-existing group dynamics formed during treatment, an important aspect of group therapy in addiction (Center for Substance Abuse Treatment, 2005). In addition, we have found that a large focus of our current addiction groups has been on the different aspects of COVID-19 and away from focusing on recovery from their addiction. The work completed in the groups then becomes containment of anxiety due to COVID-19 and deviates from focusing solely on recovery from addiction.

The increased psychological burden from COVID-19 has challenged the underlying resilience and coping strategies of our patients (Polizzi *et al.* 2020). This is especially evident in terms of maintaining recovery and avoiding relapse. Enhancing protective factors and resilience in addiction treatment centres usually follows a biopsychosocial model. In addition to considering patients for evidence-based pharmacotherapy (naltrexone, acamprosate and disulfiram) and directing patients towards effective psychological therapies (cognitive behavioural therapy and more recently acceptance and commitment therapy), improving mindfulness and strengthening social interactions to provide support in times of stress and possible relapse are important (Alim *et al.* 2012).

For many years, we have utilised therapeutic leave from the hospital to improve the resilience of our inpatient group (Barlow & Dickens, 2018). This involves careful planning of a number of weekends outside the hospital, where programme participants learn to implement and practise skills obtained during their treatment (such as mindfulness, acceptance and tolerance of cravings) to maintain abstinence. The opportunity to review and support patients as they navigate this challenge is a key component of our rehabilitation programme (Walker *et al.* 2013). Given the restrictions in place due to COVID-19, this integral part of our service is no longer possible. Patients are now moving from spending a long period of time in a protected environment to an increasingly stressful outside environment with less close supports available. To compensate, additional mindfulness-based groups have been added to our programme, and more focus has been placed on planning for discharge including the use of internet-based supports (such as teleconferenced AA meetings) and practise of the social distancing and hygiene measures needed in the community.

From the outpatient perspective, social distancing measures require that, in all but the most urgent circumstances, outpatients in our service switch to a telephone consultation service. Video-based telepsychiatry would be the ideal solution to this issue (Hollander & Carr, 2020), but given the relatively quick restrictions placed on services, having the necessary equipment and training in place was not possible. Consultations via telephone can have similar efficacy to face-to-face consultations but patients can feel less supported and encouraged compared to their face-to-face counterparts (Urness *et al.* 2006). This is of particular importance in addiction during this pandemic, where external support structures to maintain recovery will be less accessible. There is concern from clinicians that given the rapid nature of this change and the loss of face-to-face assessments, patient safety could be compromised as a result (Cowan *et al.* 2019). A flexible approach to patient assessment is required despite the concerns related to COVID-19 (Marsden *et al.* 2020). Our service is still conducting face-to-face assessments for patients who require immediate review, but a number of precautionary measures are in place. Screening questionnaires for symptoms of COVID-19 are used prior to the patient attending the appointment, facemasks are worn during review and social distancing is maintained at all times.

Despite the overall negative effect that the COVID-19 restrictions have had on our service, there have been opportunities to re-evaluate existing processes in our service, to reskill in certain areas and introduce new processes that may benefit patients in the long term. One such major adaptation has been the treatment of our alcohol-dependent patients with vitamin B1 (thiamine) supplementation, an essential part of patient care given the risks of Wernicke’s encephalopathy in this cohort (Isenberg-Grzeda *et al.* 2012). Originally, all patients with severe alcohol dependence requiring prophylaxis for Wernicke’s encephalopathy would receive thiamine intramuscularly, and all patients requiring treatment for Wernicke’s encephalopathy would be transferred to a local hospital for intravenous treatment with thiamine. There are doubts about the bioavailability of the intramuscular form of thiamine compared to the intravenous form (Thomson & Marshall, 2013), hence the reason for transfer in those requiring treatment for Wernicke’s encephalopathy. We introduced the use of intravenous Pabrinex to our service for patients requiring treatment for Wernicke’s encephalopathy to reduce the transfer of patients to the acute hospital setting which potentially increases their risk of infection with COVID-19. This also reduces the risk of introducing infection into the specialist addiction setting after the patient has transferred back to our service from the acute hospital setting. The addition of this new treatment provided a challenge to our service as it required multiple disciplines to engage and support a new change. This tested the resilience of our service to adapt to change (Fridell *et al.* 2020), but ultimately it provided an opportunity for us to introduce an improved evidence-based treatment for our patients.

Another adaptation of our service has been the increased autonomy given to patients to manage aspects of their own care to adhere to social distancing guidelines. One example of this is supplying our alcohol-dependent inpatients with their own supplies of oral thiamine and multivitamin medications. Previously, the nursing staff had dispensed these medications up to four times per day, involving separate face-to-face interactions with patients. To reduce this level of interaction, patients were supplied with their own monthly supply of thiamine and multivitamin with detailed instructions on administration combined with daily reminders from the nursing staff. This change may result in decreased compliance with medication but offers the opportunity to improve patient autonomy, which has shown to be beneficial in addiction treatment (Parker *et al.* 1979).

Once the COVID-19 pandemic has passed and restrictions on practice have been lifted, it is important to maintain the lessons learned from this experience in relation to service provision. Telepsychiatry infrastructure should be incorporated and expanded in the current service, providing patients with a blended approach to patient consultation (Wind *et al.* 2020). Our experience suggests that some patients will continue to embrace digital platforms and likely rate this mode of interaction as their preferred way of interacting. Other patients have not taken to the new form of communication in a positive way, indicating their wish to attend individual and group meetings in person. Any innovations and processes occurring from the COVID-19 pandemic that deliver an improved level of care to patients (such as the use of intravenous Pabrinex in our service) should be retained and expanded upon.

## Conclusion

Addiction psychiatry faces an increased challenge during the COVID-19 pandemic to provide a high standard of care to a vulnerable population in a testing time for all individuals. The social distancing required to curb the pandemic provides an opportunity for the proliferation of addictive behaviour. Addiction services are required to adapt to COVID-19 restrictions while maintaining a high quality of care. This can involve taking on new responsibilities to manage this patient cohort and adapt to existing practices.

## Financial Support

No financial support was provided for this work.

## Conflict of interest

DC has no conflict of interest to disclose. RH has no conflict of interest to disclose. CO has no conflict of interest to disclose.

## Ethical Standards

The authors assert that all procedures contributing to this work comply with the ethical standards of the relevant national and institutional committee on human experimentation with the Helsinki Declaration of 1975, as revised in 2008.
